# SARS-associated Coronavirus Transmission, United States

**DOI:** 10.3201/eid1002.030734

**Published:** 2004-02

**Authors:** Elmira T. Isakbaeva, Nino Khetsuriani, R. Suzanne Beard, Angela Peck, Dean Erdman, Stephan S. Monroe, Suxiang Tong, Thomas G. Ksiazek, Sara Lowther, Indra Pandya Smith, Larry J. Anderson, Jairam Lingappa, Marc-Alain Widdowson, other members of the SARS Investigation Group

**Affiliations:** * Centers for Disease Control and Prevention, Atlanta, Georgia, USA; † McKing Consulting, Atlanta, Georgia, USA

**Keywords:** severe acute respiratory syndrome (SARS), outbreak, SARS-associated coronavirus, epidemiology, transmission, natural history

## Abstract

To better assess the risk for transmission of the severe acute respiratory syndrome–associated coronavirus (SARS-CoV), we obtained serial specimens and clinical and exposure data from seven confirmed U.S. SARS patients and their 10 household contacts. SARS-CoV was detected in a day-14 sputum specimen from one case-patient and in five stool specimens from two case-patients. In one case-patient, SARS-CoV persisted in stool for at least 26 days after symptom onset. The highest amounts of virus were in the day-14 sputum sample and a day-14 stool sample. Residual respiratory symptoms were still present in recovered SARS case-patients 2 months after illness onset. Possible transmission of SARS-CoV occurred in one household contact, but this person had also traveled to a SARS-affected area. The data suggest that SARS-CoV is not always transmitted efficiently. Laboratory diagnosis of SARS-CoV infection is difficult; thus, sputum and stool specimens should be included in the diagnostic work-up for SARS-CoV infection.

Severe acute respiratory syndrome (SARS) was recently described as the clinical manifestation of infection by a novel coronavirus (CoV), the SARS-associated CoV (SARS-CoV) (*1*–*5*). This syndrome was first recognized in February 2003 in Vietnam, but it was later realized that the first cases occurred in southern China in November 2002 (*6*,*7*). Subsequently, the infection rapidly spread throughout the world, and by July 2003, when the World Health Organization declared that the outbreak was contained, 8,437 cases and 813 deaths in 32 countries had been reported (*8*).

As the outbreak developed, epidemiologic evidence suggested that SARS-CoV was transmitted by respiratory droplets or direct contact with infected patients and possibly by fomites (*9*–*12*). In certain circumstances, transmission of SARS-CoV was particularly efficient and resulted in individual patients infecting large numbers of people (referred to as “super-spreading events”), whereas in other situations, no secondary transmission was observed (*13*).

A better understanding of the duration of SARS-CoV shedding and virus quantities in respiratory secretions, stool, urine, and other body fluids and of the risk factors for spreading illness to close contacts is critical to accurately assess the risk for transmission and to develop effective control strategies. To that end, we obtained serial biologic specimens and clinical and exposure data for 5 to 10 weeks after onset of illness from seven laboratory-confirmed U.S. SARS patients and their household contacts.

## Materials and Methods

### Participants

We targeted 103 patients who met the Centers for Disease Control and Prevention’s (CDC’s) surveillance case definition for probable SARS (*14*). Of these patients, 7 (7%) with laboratory-confirmed SARS-CoV infection (antibodies to SARS-CoV were detected) were enrolled; 19 (18%), including 1 confirmed SARS case-patient, declined participation; and 77 (75%) were excluded for various reasons (negative for SARS-CoV antibody at ≥21 days after illness onset, a confirmed alternative diagnosis, or foreign citizen not residing in the United States). The household contacts of seven laboratory-confirmed case-patients were also enrolled. Household contacts were defined as persons who had lived in the same household with SARS case-patients during their illness. All participants provided informed consent.

### Timeline for Follow-up Visits

Follow-up visits were scheduled twice a week for the first 3 weeks after illness onset and then once a week for 2 weeks thereafter. If a case-patient was first enrolled after week 5 of illness, then a follow-up visit was made as soon as feasible after enrollment. Some case-patients were enrolled at >5 weeks after illness onset and, therefore, were followed up for >10 weeks. For household contacts, visits were scheduled once weekly for a period of 4 weeks after initial exposure to the case-patient. A single follow-up visit was scheduled if the household contact was enrolled >4 weeks after initial exposure to the case-patient.

### Clinical and Epidemiologic Data

At the initial visit with the SARS case-patients, we collected data on demographics, date of illness onset, clinical symptoms, and exposure history. At the initial visit with household contacts, we gathered data on any illness they had had since their exposure to the case-patient and on the types and patterns of exposure (e.g., sleeping in the same room at night, daily contact within <3 feet, and direct skin-to-skin contact, such as kissing or hugging, with case-patients). At each subsequent visit, we collected information on any symptoms experienced by case-patients or household contacts since their previous visit, including symptoms during the current visit.

### Clinical Specimens

Specimens collected as a part of the diagnostic work-up were available for this investigation, and at each postenrollment visit, participants were asked to provide whole-blood, serum, stool, urine, nasopharyngeal, and oropharyngeal swab specimens. We obtained 1–10 mL of blood from adults and 0.5–5 mL of blood from children <3 years old by venipuncture or finger stick. Clotted blood was centrifuged, and serum was separated before being shipped to CDC for testing. Similar volumes of whole blood were collected in a tube containing EDTA. Nasopharyngeal and oropharyngeal samples were collected by use of a single Dacron swab with a nonwooden shaft; the swab was then placed in a sterile vial containing 2 mL of viral transport medium. Stool specimens were collected in a sterile container and sealed. Participants provided a 50-mL clean-catch collection of urine in a sterile urine cup. Specimens were processed and stored according to CDC laboratory biosafety guidelines (*15*). All specimens were stored at 4°C for a maximum of 72 h and shipped on ice to the CDC laboratory. If shipping within 72 h was not feasible, specimens were stored at –70°C and then shipped.

### Laboratory Methods

To detect SARS-CoV in stool, urine, and respiratory specimens, we performed reverse transcriptase–polymerase chain reaction (RT-PCR), using primers targeted to the polymerase and nucleocapsid genes of the SARS-CoV genome, as described elsewhere (2, Emery et al., unpub data). Stool samples were prepared as 10% extracts in Tris-HCl buffer before isolation of total nucleic acid for RT-PCR testing. To quantify the virus load in respiratory and stool specimens, quantitative RT-PCR was performed using the **TaqMan** assay a**nd** standard curves generated from synthetic RNA transcripts (S. S. Monroe & R. S. Beard, unpub. data). Previously described culture techniques (*2*) were used to isolate SARS-CoV from specimens. To determine the S and N gene sequences of SARS-CoV, a set of 10 overlapping RT-PCR products, which cover the entire open reading frames of the S (8 products) and N (2 products) genes, were generated by using the SuperScript One-Step RT-PCR with Platinum Taq (Invitrogen, Carlsbad, CA) and sequenced by using 16 (S gene) or 7 (N gene) sequencing primers (S. Tong et al, unpub. data). Serum specimens were tested for SARS-CoV–associated antibodies by use of an enzyme-linked immunosorbent assay and an indirect fluorescent antibody test, using previously described methods (*2*). Serum specimens were considered positive only if results for both tests were positive using predetermined cut-offs (*2*).

## Results

### Follow-up Findings

Five of seven enrolled case-patients provided data on residual symptoms. Three case-patients reported shortness of breath that persisted at least until days 50, 56, and 62, respectively, after onset of fever. Two case-patients reported residual coughing: case-patient 4 reported a dry cough until day 50 and case-patient 2 reported a productive cough until day 56 after onset of fever. These symptoms had been reported during the acute phase of each case-patient’s illness. Wheezing developed in one case-patient without a previous history of respiratory disease at day 11 of illness and persisted at least until day 46. No data were available to characterize the progression of symptoms over time.

Of 41 respiratory specimens obtained from seven case-patients (Table 1), 4 (10%) were sputum samples from two case-patients (1 from case-patient 5 and 3 from case-patient 7). SARS-CoV was detected by both RT-PCR and viral culture in the sputum sample of case-patient 5, which was collected at day 14 after illness onset (Figure). All other respiratory specimens, including seven nasopharyngeal and oropharyngeal swab samples collected during the first 2 weeks of illness from five case-patients, tested negative by RT-PCR.

**Table 1:** Timing of collection of clinical specimens from seven confirmed SARS case-patients, United States, 2003^a^

Specimen type	No. of specimens (no. of case-patients) by no. of days after illness onset			Total no. of specimens
	0–14 days	15–28 days	>28 days	
Respiratory	11 (7)	12 (4)	18 (7)	41
Sputum	2 (2)	2 (1)	0 (0)	4
NP swab	5 (5)	4 (4)	9 (6)	18
OP swab	2 (2)	6 (4)	9 (7)	17
Nasal aspirate	1 (1)	0 (0)	0 (0)	1
Nasal wash	1 (1)	0 (0)	0 (0)	1
Stool	1 (1)	5 (2)	8 (6)	14
Urine	0	2 (2)	6 (5)	8
Serum/blood	18 (7)	15 (4)	15 (7)	48

^a^SARS, severe acute respiratory syndrome; NP, nasopharyngeal; OP, oropharyngeal.

**Figure:**
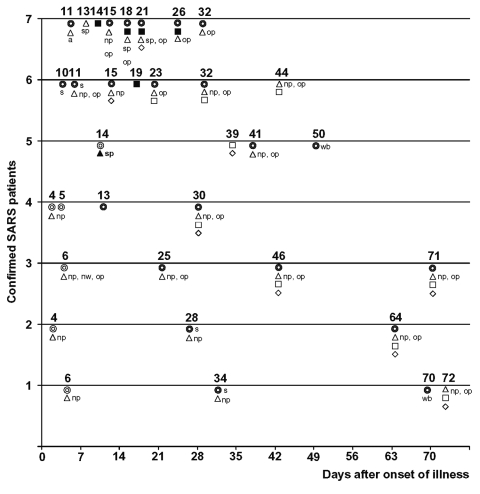
Detecting severe acute respiratory syndrome–associated coronavirus (SARS-CoV) RNA by reverse transcriptase–polymerase chain reaction (RT-PCR) and SARS-CoV antibodies by enzyme-linked immunosorbent assay (ELISA) in clinical specimens from seven confirmed SARS case-patients, United States, 2003. Circle within circle: blood specimens (same symbol represents both whole blood and serum when both specimens are collected and results are entirely concordant. s, serum; wb, whole blood (symbols are labeled s or wb if either blood or serum was collected). Blocked symbols denote SARS-CoV–positive specimens by ELISA. **∆**: respiratory specimens (include np, nasopharyngeal swab; nw, nasal wash; a, nasal aspirate; op, oropharyngeal swab; sp, sputum). □: stool. ◊: urine. Blocked symbols denote SARS-CoV–positive specimens by RT-PCR.

A total of 14 stool specimens were obtained from seven case-patients: two patients provided 4 samples each, one patient had 2 samples, and four had 1 sample each. SARS-CoV RNA was detected in five specimens, all of which came from two case-patients (one specimen from case-patient 6 and four specimens from case-patient 7) (Figure). The single positive stool specimen from case-patient 6 was obtained 19 days after onset; his subsequent stool specimens (collected at days 23, 32, and 44) tested negative for SARS-CoV by RT-PCR. The first stool specimen from case-patient 7 was collected on day 14 of illness; viral RNA was detected in all four of his stools, including the last one, which was collected at day 26. SARS-CoV was not isolated by culture from any of the RT-PCR–positive stool specimens.

The highest concentrations of SARS-CoV were detected in sputum from case-patient 5 (43 million copies per gram of specimen) and in the day-14 stool from case-patient 7 (37 million copies per gram of specimen) (Table 2). After day 14 of illness, the concentration of virus in stool specimens from case-patient 7 dropped by 20-fold or more. Of note, this case-patient reported moderate diarrhea from days 2 to 12 of illness. Case-patient 6 had only mild diarrhea during the first 4 days of illness, and the amount of virus in his stool sample that was collected on day 19 (i.e., 2 weeks after the resolution of diarrhea) was approximately 800-fold lower than the amount in the day-14 stool sample of case-patient 7 and approximately 50-fold lower than that found in subsequent specimens from case-patient 7. No evidence was found that the virus mutated in case-patient 7 during the infection: genomic sequences of **S and N genes** of SARS-CoV from all positive stool specimens of this case-patient were identical.

**Table 2:** Quantities of SARS-CoV in sputum and stool specimens from three confirmed SARS case-patients, as measured by quantitative RT-PCR, United States, 2003^a^

Case-patient identification no.	Specimen	Time of specimen collection after illness onset (no. of days)	Copies per gram of sample
5	Sputum	14	43,000,000
7	Stool	14	37,000,000
	Stool	18	1,600,000
	Stool	21	930,000
	Stool	26	2,300,000
6	Stool	19	45,000

^a^SARS-CoV, severe acute respiratory syndrome–associated coronavirus; RT-PCR, reverse transcriptase–polymerase chain reaction.

No viral RNA was detected by RT-PCR in any of the eight urine specimens collected from the seven case-patients. SARS-CoV antibody was first found as early as days 10 and 11 after illness onset in three of seven case-patients. Adequate specimens were not available to characterize the time of first detectable SARS-CoV antibody in the remaining four case-patients (Table 3).

**Table 3:** SARS-CoV antibodies as determined by enzyme-linked immunosorbent assay in seven confirmed SARS case-patients, by number of days after illness onset, United States, 2003^a^

Case-patient	Days after illness onset	SARS-CoV antibodies^b^
Patient 1		
	6	Negative
	34	1,600
Patient 2		
	4	Negative
	28	6,400
	64	6,400
Patient 3		
	6	Negative
	25	6,400
	46	1,600
	71	1,600
Patient 4		
	2	Negative
	5	Negative
	13	Negative
	30	6,400
Patient 5		
	14	Negative
	41	1,600
Patient 6		
	10	1,600
	11	1,600
	15	6,400
	23	6,400
Patient 7		
	11	400
	15	1,600
	18	6,400
	21	6,400
	6	1,600
	32	6,400

^a^SARS-CoV, severe acute respiratory syndrome–associated coronavirus. ^b^Reciprocal of dilution.

### Household Transmission

Ten household contacts of five of the seven SARS case-patients were enrolled. Case-patient 1 had four household contacts, case-patients 3 and 4 had one such contact each, and case-patients 6 and 7 had two household contacts each. Of the 10 household contacts, 4 were female and 6 were male, 2 were smokers, and 2 reported previous history of respiratory problems (sarcoidosis in household contact 5 and pulmonary embolus in household contact 7).

Household contact 1 (who was also case-patient 2) was the only such contact who tested positive for SARS-CoV antibody. The remaining nine household contacts were negative for SARS-CoV antibody in specimens collected >28 days after their initial exposure to a case-patient. The infected household contact was the wife of confirmed SARS case-patient 1. The couple had visited Hong Kong together in early March 2003 and stayed at Hotel M, which was subsequently linked to the initial spread of SARS (*16*), where they had multiple opportunities for exposure. Case-patient 1 became ill 7 days after returning to the United States from Hong Kong. Symptoms developed in household contact 1 some 13 days after returning to the United States and 6 days after onset of illness in her husband. SARS-like symptoms did not develop in any of the three other household contacts of case-patient 1, nor did any have laboratory evidence of SARS-CoV infection. The analysis of household exposures and protective measures in this household indicated that household contact 1 had more frequent unprotected contact with the index patient compared with three other household contacts (Table 4).

**Table 4:** Profile and exposure of 10 household contacts (HHCs) of five confirmed SARS case-patients, United States, 2003^a^

HHC no.	Case-patient identification no. (n=5)	Shedding documented in case-patient	Use of surgical mask by case-patient	SARS-CoV infection in HHC	HHC relation to case-patient	Age (y)/sex/race	Exposure to the case-patient before hospitalization				Protective measures by HHC	
							No. of days in house with case-patient	No. of nights in same room	Contact within 3 feet (h/day)	Skin-to-skin contact (times/day)	Surgical mask used during 1st week of illness	Routine handwashing with soap
1^b^	1	No	No	Yes	Spouse	37/F/A	4	5–6	0–1	>3	No	No^c^
2				No	Brother	57/M/A	4	0	1–3	0	No	No^c^
3				No	Brother-in-law	55/M/A	4	0	0–1	0	No	Yes
4				No	Nephew	16/M/A	4	0	0–1	0	No	Yes
5	3	No	No	No	Spouse	52/M/W	6	7	>7	>3	No	Yes
6	4	No	No	No	Mother	52/F/W	4	0	>7	>3	No	Yes
7	6^d^	Yes	Yes	No	Spouse	47/F/W	All^e^	0	0–1	1–2	Yes	Yes
8				No	Son	12/M/W	All^e^	0	1–3	1–2	No	Yes
9	7^f^	Yes	Yes	No	Son	22/M/A	11	0	0–1	1–2	Yes	Yes
10				No	Daughter	15/F/A	11	0	0–1	0	Yes	Yes

^a^SARS, severe acute respiratory syndrome; F, female; M, male; A, Asian; W, white. ^b^SARS coronavirus antibody–positive HHC. ^c^No soap used for handwashing (water only). ^d^Shedding documented in stool on day 19 after onset of illness. ^e^Case-patient 6 was never hospitalized. fShedding documented in stool on days 14, 18, 21, and 26 after onset of illness.

The remaining six uninfected household contacts reported close contact (e.g., contact within 3 feet and unprotected skin-to-skin contact) with case-patients. The exposure of four household contacts of two case-patients with stool specimens positive for SARS-CoV was limited by isolation of the case-patients in a separate room with a private bathroom during the first week of illness. Both case-patients also wore surgical masks during this period, as did three of their four household contacts. Case-patient 7 was hospitalized from day 11 to day 18 of illness, the period during which the highest amounts of virus were detected in his stool, and continued to be positive for SARS-CoV in stool after discharge. Neither case-patient 7 nor his two household contacts wore surgical masks after being discharged from the hospital. Case-patient 6, who was never hospitalized, had low-level shedding of SARS-CoV in stool on day 19, but no virus was subsequently found in his stool specimens. One of his two household contacts wore a mask until 10 days after the resolution of fever in the case-patient.

## Discussion

In this investigation of U.S. SARS-CoV–infected persons and their household contacts, we identified probable transmission of SARS-CoV to only 1 of 10 such contacts. We detected SARS-CoV in fecal and respiratory specimens and found that SARS case-patients may have high concentrations of virus in stools during the 2nd week of illness and continue to shed the virus in feces until at least 26 days after onset of symptoms. The amount of SARS-CoV in stool from a case-patient with moderate diarrhea was similarly high to the quantity seen in a sputum specimen collected from a different case-patient at the same interval after illness onset. However, no virus could be cultured from any stool specimens that were PCR-positive for SARS-CoV, suggesting that SARS-CoV in feces may be present in the form of either nonviable viral particles or antibody-coated virus.

The one household contact who became infected was the person who had more contact with the potential source case-patient during the first week of illness than did other members in the household. This contact was also exposed in Hong Kong along with her husband; however, she became ill >10 days after returning to the United States (*16*). Previously reported data suggest that the incubation period for SARS ranges from 2 to 10 days (*4*,*17*), but in some cases, the incubation period may be as long as 14 days (*18*). Therefore, the possibility remains that this contact may have been infected in Hong Kong. The remaining uninfected household contacts included four contacts of two case-patients with positive stool specimens in whose households simple infection-control procedures were implemented during the acute phase of illness in the index patient.

The lack of widespread household transmission of SARS found in our investigation is similar to findings in reports of the outbreak in Toronto, where 2 (6%) of 33 household contacts were infected despite unprotected contact with a SARS case-patient (*19*), and from the Philippines, where <1% of nonhospital contacts were reported to be infected (*20*). This finding supports the idea that in certain circumstances, SARS-CoV is not easily transmitted. Transmission may also be more likely to occur at the time when patients are shedding higher amounts of virus, and this period may coincide with their hospitalization, thus decreasing the degree of exposure for household contacts.

We were unable to detect SARS-CoV in specimens of our case-patients before day 14 after illness onset. We only detected virus in three case-patients: in a sputum sample of one patient at day 14 and in stool samples of two patients at day >14. All upper respiratory specimens in the first 2 weeks after onset were negative for SARS-CoV by RT-PCR; this finding differs from a report in Hong Kong, where viral RNA was detected in nasopharyngeal aspirates of 68% of case-patients at day 14 (*21*). Our inability to detect the virus in early respiratory samples may be associated with the type (nasopharyngeal and oropharyngeal swabs versus nasopharyngeal aspirates) of collected specimens, as well as with low amounts of virus generally seen in such specimens (*1*). Sputum samples may have a higher concentration of virus than upper respiratory specimens (*1*), consistent with our findings. Stool specimens have been found positive more frequently than upper respiratory specimens during the 2nd and 3rd week of illness, which is in accord with the limited results of this study. The inability to detect SARS-CoV in urine may be the result of a late collection of urine specimens (>14 days after illness onset). A wider use of steroids in treatment of case-patients in Hong Kong compared with case-patients in the United States may have also altered the pattern of shedding of SARS-CoV.

Persistent respiratory symptoms that were reported up until at least 2 months after onset by most of our case-patients were similar to symptoms observed by Avendano et al. (*19*) in a study of Canadian healthcare workers who were followed for 5 weeks after illness onset, suggesting residual illness in SARS case-patients. However, the progression of these symptoms over time is difficult to interpret without a better appreciation of the pre-illness symptoms. Antibody to SARS-CoV in some case-patients was documented as early as day 10 after illness onset. We did not have an adequate number of early serum specimens from other case-patients to determine when SARS-CoV antibody is first detectable.

Results of this investigation should be interpreted in light of several limitations. The small number of participants does not allow for accurate estimation of the risk for transmission to household members. Irregular and long intervals between collections of specimens do not permit a clear picture of the natural history of SARS-CoV infection, including documenting the precise timing of the first appearance of SARS-CoV antibody. We also may have missed the presence of shedding in stools of other case-patients who had reported diarrhea during the acute phase of illness. Possible variations in specimen collection and handling techniques could also have affected SARS-CoV detection rates in respiratory and stool specimens.

Our results suggest that SARS-CoV is not always transmitted efficiently. The early laboratory diagnosis of SARS-CoV infection is difficult; optimal timing for specimen collection and currently available diagnostic tests need to be refined further. Collecting and testing stool and sputum should be included as a part of the diagnostic work-up for SARS-CoV infection. A follow-up of recovered SARS case-patients over several months would also help to better assess possible waning of antibody titers and long-term sequelae of the disease and, thus, improve our understanding of the true illness associated with SARS-CoV infection.

## References

[1] Drosten C, Gunther S, Preiser W, van der Werf S, Brodt HR, Becker S, Identification of a novel coronavirus in patients with severe acute respiratory syndrome. N Engl J Med. 2003;348:1967–76. 10.1056/NEJMoa030747 12690091

[2] Ksiazek TG, Erdman D, Goldsmith CS, Zaki SR, Peret T, Emery S, A novel coronavirus associated with severe acute respiratory syndrome. N Engl J Med. 2003;348:1953–66. 10.1056/NEJMoa030781 12690092

[3] Peiris JS, Lai ST, Poon LL, Guan Y, Yam LY, Lim W, Coronavirus as a possible cause of severe acute respiratory syndrome. Lancet. 2003;361:1319–25. 10.1016/S0140-6736(03)13077-2 12711465 PMC7112372

[4] Lee N, Hui D, Wu A, Chan P, Cameron P, Joynt GM, A major outbreak of severe acute respiratory syndrome in Hong Kong. N Engl J Med. 2003;348:1986–94. 10.1056/NEJMoa030685 12682352

[5] Poutanen SM, Low DE, Henry B, Finkelstein S, Rose D, Green K, Identification of severe acute respiratory syndrome in Canada. N Engl J Med. 2003;348:1995–2005. 10.1056/NEJMoa030634 12671061

[6] World Health Organization. Acute respiratory syndrome. China, Hong Kong Special Administrative Region of China, and Viet Nam. Wkly Epidemiol Rec 2003;78:73–4. Available from: URL: http://www.who.int/wer/pdf/2003/wer7811.pdf 12674023

[7] World Health Organization. Update 95—SARS: Chronology of a serial killer. Communicable Disease Surveillance and Response (CSR). Available from: URL: http://www.who.int/csr/don/2003_07_04/en/

[8] World Health Organization. Cumulative number of reported probable cases of SARS. Communicable Disease Surveillance and Response (CSR). Available from: URL: http://www.who.int/csr/sars/country/2003_07_11/en/

[9] Centers for Disease Control and Prevention. Cluster of severe acute respiratory syndrome cases among protected health care workers—Toronto, April 2003. MMWR Morb Mortal Wkly Rep. 2003;52:433–6. 12807083

[10] Tsang KW, Ho PL, Ooi GC, Yee WK, Wang T, Chan-Yeung M, A cluster of cases of severe acute respiratory syndrome in Hong Kong. N Engl J Med. 2003;348:1977–85. 10.1056/NEJMoa030666 12671062

[11] Department of Health, Government of Hong Kong Special Administrative Region. Outbreak of severe acute respiratory syndrome (SARS) at Amoy Gardens, Kowloon Bay, Hong Kong: main findings of the investigation. April 17, 2003. Available from: URL: http://www.info.gov.hk/info/ap/pdf/amoy_e.pdf

[12] Booth CM, Matukas LM, Tomlinson GA, Rachlis AR, Rose DB, Dwosh HA, Clinical features and short-term outcomes of 144 patients with SARS in the greater Toronto area. JAMA. 2003;289:2801–9. 10.1001/jama.289.21.JOC30885 12734147

[13] Centers for Disease Control and Prevention. Severe acute respiratory syndrome—Singapore, 2003. MMWR Morb Mortal Wkly Rep. 2003;52:405–11. 12807088

[14] Centers for Disease Control and Prevention. Updated interim surveillance case definition for severe acute respiratory syndrome (SARS)—United States, April 29, 2003. MMWR Morb Mortal Wkly Rep. 2003;52:391–3. 12765204

[15] Centers for Disease Control and Prevention. **Interim laboratory biosafety guidelines for handling and processing specimens associated with severe acute respiratory syndrome (SARS). August 18,** 2003**. Available from: URL:** http://www.cdc.gov/ncidod/sars/sarslabguide.htm

[16] Centers for Disease Control and Prevention. Update: outbreak of severe acute respiratory syndrome—worldwide, 2003. MMWR Morb Mortal Wkly Rep. 2003;52:241–8. 12680518

[17] Varia M, Wilson S, Sarwal S, McGeer A, Gournis E, Galanis E, Investigation of a nosocomial outbreak of severe acute respiratory syndrome (SARS) in Toronto, Canada. CMAJ. 2003;169:285–92. 12925421 PMC180651

[18] Donnelly CA, Ghani AC, Leung GM, Hedley AJ, Fraser C, Riley S, Epidemiological determinants of spread of causal agent of severe acute respiratory syndrome in Hong Kong. Lancet. 2003;361:1761–6. 10.1016/S0140-6736(03)13410-1 12781533 PMC7112380

[19] Avendano M, Derkach P, Swan S. Clinical course and management of SARS in health care workers in Toronto: a case series. CMAJ. 2003;168:1649–60. 12821618 PMC161610

[20] SARS outbreak in the Philippines. Wkly Epidemiol Rec 2003;78:189–96. Available from: URL: http://www.who.int/docstore/wer/pdf/2003/wer7822.pdf 12836452

[21] Peiris JS, Chu CM, Cheng VC, Chan KS, Hung IF, Poon LL, Clinical progression and viral load in a community outbreak of coronavirus-associated SARS pneumonia: a prospective investigation. Lancet. 2003;361:1767–72. 10.1016/S0140-6736(03)13412-5 12781535 PMC7112410

